# Relationships between catastrophic thought, bodily sensations and physical symptoms

**DOI:** 10.1186/s13030-017-0110-z

**Published:** 2017-11-08

**Authors:** Hiroshi Seto, Mutsuhiro Nakao

**Affiliations:** 1 Student Support Office, Iwate Prefectural University, 152-52, Sugo, Takizawa-shi, Iwate, 020-0693 Japan; 2 Teikyo Graduate School of Public Health & Division of Psychosomatic Medicine, Teikyo University Hospital, 2-11-1, Kaga, Itabashi-ku, Tokyo, 173-8605 Japan

**Keywords:** Physical symptoms, Somatosensory amplification, Somatosensory catastrophizing

## Abstract

**Background:**

Researchers have recently begun to seek cognitive explanations for physical symptoms with no obvious biological cause. Concepts such as somatization, somatosensory amplification, and somatosensory catastrophizing have been invoked to explain these phenomena. Somatosensory amplification occurs when these bodily sensations become stronger and more painful. Somatosensory catastrophizing is the tendency to attribute these bodily sensations to unbearable functional modulation or as signs of serious illness. This causes the sufferer to pay excessive attention to these physical sensations. However, there is no scale for evaluating somatosensory catastrophizing, and there are no standard diagnostic criteria. There were two objectives for this study: to develop a scale for evaluating somatosensory catastrophizing and to investigate relationships between somatosensory amplification, somatosensory catastrophizing, and physical symptoms.

**Methods:**

In the first part of this study, in which we developed the scale, there were 231 student participants with an average age of 20.1 years. Of these, 57% of the participants were female. In the second part of the study, there were two groups of participants. The first group consisted of 158 non-patient subjects, 56% of whom were female, with an average age of 20.2 years. There were 33 outpatients receiving treatment for somatoform disorders in the second group. The average age of these participants, of whom 67% were female, was 48.8 years. The second part of the study was conducted using standardized self-rating questionnaires to assess somatosensory amplification and catastrophizing.

**Results:**

We developed a 27-item scale, which we have called the Somatosensory catastrophizing scale (SSCS). The SSCS assesses five key areas, and our analysis confirmed it to be valid and highly reliable. The scale identified that the patient group from the second part of the study scored more highly than the control group for both somatosensory amplification and catastrophizing. Additionally, the results of covariance structure analyses revealed a significant causal relationship of the form “somatosensory amplifcation” via “somatosensory catastrophizing” to “physical symptoms”. This relationship held in both groups of participants. The key difference between the patient and non-patient groups was that somatosensory catastrophizing had a greater impact on the physical symptoms of the participants in the patient group.

**Conclusions:**

In this study, we developed the SSCS, which enables us to measure somatosensory catastrophizing empirically. We then clarified the relationship between somatosensory amplification, somatosensory catastrophizing, and physical symptoms. In the future, we expect to be able to apply our new understanding to developing intervention techniques to mitigate the physical symptoms caused by somatosensory catastrophizing.

**Electronic supplementary material:**

The online version of this article (doi:10.1186/s13030-017-0110-z) contains supplementary material, which is available to authorized users.

## Background

The concept of somatosensory amplification has been developed to explain physical symptoms that cannot be explained by any obvious biological mechanisms, such as functional body syndromes. Somatosensory amplification is the tendency to experience strong bodily sensations, to the extent that they become harmful and troublesome [1]. Specifically, somatosensory amplification is characterized by excessive arousal; a tendency to selectively pay attention to specific bodily sensations even though they are not frequent or intense; and emotional and cognitive tendencies to acknowledge and heighten bodily sensations, causing the sufferer to be vigilant for their symptoms.

Somatosensory amplification is thought to have both a stable property from the birth and a state property, which can perceive specific sensations to a different extent in different situations. The types of bodily sensations that can be amplified include normal physiological responses such as hypotension and tachycardia when standing; shortness of breath during exercise; benign functional abnormalities such as transient tinnitus and headaches; somatosensory sensations due to tension in the sympathetic nervous system caused by pain or anxiety; and sensations caused by physical disorders, etc. [2].

Barsky, Wyshak, & Klerman [3] developed a self-contained questionnaire called the somatosensory amplification scale (SSAS) to measure the extent of somatosensory amplification (Table 1). Historically, somatosensory amplification has been studied in relation to hypochondriasis [3]_._ Barsky [2] expanded the scope of the applications of the concept of somatosensory amplification and summarized the diseases and pathological conditions that may be associated with it.

**Table 1:** Somatosensory amplification scale

1. When someone else coughs, it makes me cough too.
2. I can't stand smoke, smog, or pollutants in the air.
3. I am often aware of various things happening within my body.
4. When I bruise myself, it stays noticeable for a long time.
5. Sudden loud noises really bother me.
6. I can sometimes hear my pulse or my heartbeat throbbing in my ear.
7. I hate to be too hot or too cold.
8. I am quick to sense the hunger contractions in my stomach.
9. Even something minor, like an insect bite or a splinter, really bothers me.
10. I have a low tolerance for pain.

**Fig. 1:**
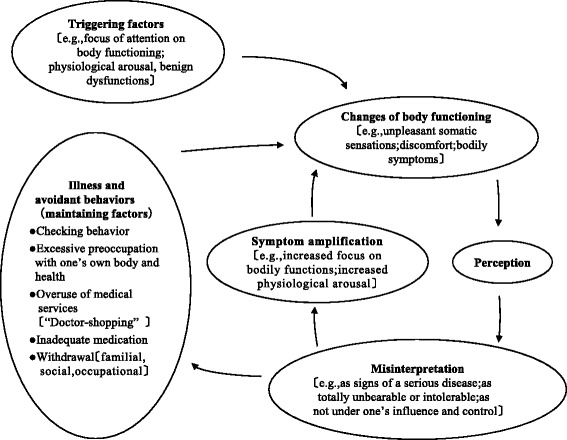
A cognitive-behavioral model of somatoform disorders

Hiller & Rief [4] proposed a cognitive-behavioral model of somatoform disorders based on cognitive behavior theory and clinical experience. They noted the relationship between a patient’s cognitive behavioral features and the symptoms of their somatoform disorder (Fig. 1). In this model, after perceiving changes in physical function accompanied by physical arousal or benign dysfunction, the patient’s symptoms worsen due to excessive interpretation. This leads to catastrophic thinking, such as feelings like “this is completely unbearable”, " this is a sign of a serious illness", and "there is nothing I can do" etc. Along with that, disease-avoidance behaviors such as confirmation action and withdrawal will occur, and symptoms will be maintained.

Hiller & Rief [4] developed an intervention method based on the above model. Their intervention aims to break the cognitive-behavioral vicious circle. They state that, first of all, it is necessary to provide the patient with conventional medical explanations and discuss the biological, psychological, and social aspects of their symptoms. They then encourage the patients to use a daily symptom diary to discover the relationships between their symptoms and factors that are easy to change such as mood, stress, and physical discomfort. Biofeedback and stress tolerance tests are helpful for improving patients’ understanding of the psychological factors associated with their symptoms, and relaxation techniques are effective for reducing physical arousal. It is also useful to identify patients’ false beliefs about their symptoms and help them to reconsider the validity of their reasoning. Furthermore, they make behavioral interventions. In particular, they highlight the importance of increasing physical activity levels, reducing disease-causing behaviors and avoiding behaviors that lead to the continuation of the vicious cycle.

Catastrophic thinking is a concept advocated by Ellis [5], the founder of rational emotive therapy, that refers to a thinking style that overestimates an event as threatening, and in recent years, research on the relationship with “pain” has been advanced. Sullivan, Bishop, & Pivik [6] have created the Pain Catastrophizing Scale (PCS) to measure the extent of catastrophic thought to pain and have studied the relationship between catastrophic thought to pain and chronic pain. According to them, when the tendency of catastrophic thought to pain is strong, the pain increases, and catastrophic thought to pain becomes a predictor about pain prognosis.

Also, in the study of individual disease states, for example, a report showed that catastrophic thinking is a factor that exacerbates and maintains chronic low back pain [7] and another showed that catastrophic thinking is more predictive than demographic variables such as physical factors, age and gender regarding chronic low back pain prognosis [8]. In addition, a report found a strong correlation between the severity of migraine and the catastrophic thought to pain [9]. In addition, for patients with tension type headache, it was reported that catastrophic thought to pain induces escape/avoidance behavior and affects the degree of difficulty in daily life [10].

Although research on the relationship between pain and catastrophic thinking has been done in this way, research on the relationship between general physical symptoms, including pain and catastrophic, thinking is not enough. Considering that a relationship between pain, a physical symptom, and catastrophic thinking is recognized, a significant relation between various physical symptoms including pain and catastrophic thought to body sensation can be presumed. In somatoform disorder, because diverse symptoms such as nausea, numbness, malaise, etc. are seen, it is necessary to examine the relation between general physical symptoms, including pain, and catastrophic thinking.

As described above, in previous study the consideration of physical symptoms was advanced from a cognitive point of view, and “catastrophic thought to body sensation” is thought to be an important concept to explain physical symptoms that can not be explained by obvious organic factors. However, there is no scale to measure the transition from catastrophic thought to body sensation. Hence, it is not possible to empirically investigate the parameters that affect this transition. Current research on the cognitive processes leading to deterioration in physical symptoms is insufficient. Therefore, we developed the SSCS and investigated the relationship between physical symptoms, somatosensory amplification, and somatosensory catastrophizing.

The purpose of the first part of this study was to determine factors that may contribute to catastrophic thoughts developing into bodily sensation and to prepare a draft of the SSCS. We based our final SSCS on the scale we developed in the preliminary research and on the results of the statistical analyses that we conducted to assess its reliability and validity. In the second part of the study, we determined the effects that somatosensory amplification and somatosensory catastrophizing have on physical symptoms by comparing a patient group who suffered from somatoform disorders with a control group. The studies were conducted with the approval of the ethics committee (reference number: Waseda Univ. Res.397). We obtained informed written consent from all of the participants.

## Methods

### First part of the study

A total of 231 university students participated in the preliminary investigation. Their average age was 20.1 years and 57% of them were female. They responded to our questions in free prose. We asked them about the bodily sensations they routinely perceive and the nature of the catastrophic thoughts that contribute to the bodily sensations. We used the KJ method [11] to classify and organize the results.

In the main investigation, we asked 158 university students to answer questions relating to the original scale. The questions were grouped into five subject areas. The average age of the participants in this group was 20.2 years, and 56% of them were female. We used the pain catastrophizing scale (PCS) [6] and the short health anxiety inventory (SHAI) [12] to assess the validity of the SSCS.

### Second part of the study

We recruited 33 outpatients with an average age of 48.8 years, 67% of whom were female, to the patient group. These patients had been diagnosed with somatoform disorders by doctors from the department of psychosomatic medicine at the university hospital. There were 123 university students in the control group. Their average age was 20.5 and 72% of them were female. They completed three surveys: 1) the SSCS prepared in the first part of this study 2) the SSAS, 3) the medical symptom checklist (MSC) [13, 14], which was used to evaluate the participants’ physical symptoms.

## Results

### First part of the study

We identified 38 items to potentially include in the SSCS in the preliminary investigation (Additional file 1: Appendix). Of these, we excluded items that we did not consider to be catastrophic thoughts and items that were too similar to each other. We also adopted two items from an existing related scale, the PCS. This resulted in an original scale comprising 29 items.

In our main investigation, we assumed that the items on our scale were correlated. Hence, we performed factor analysis based on promax rotation. We extracted items with a factor load greater than or equal to 40. This left us with 27 items grouped into five areas: attention to bodily sensation, obstacles to daily life, concerns about serious disease, feeling helpless to do anything about the symptoms, and hopelessness. To determine whether the resulting scale was valid, we calculated Cronbach’s alpha. This was greater than 0.90 for the first to the third factors, and greater than 0.85 for the fourth and fifth factors. We then calculated the Pearson’s correlation coefficients of the SSCS, PCS and SHAI to establish their concurrent validity. There was a strong positive correlation (*r* = .76, *p* < .01) between the SSCS and the PCS and a relatively strong positive correlation (*r* = .61, *p* < .01) between the SSCS and the SHAI. Hence, we produced a valid, reliable SSCS that covers five areas by asking about 27 items, which are assessed using a five-point Likert scale. The items in the SSCS and the results of the factor analysis are shown in Table 2.

**Table 2:**
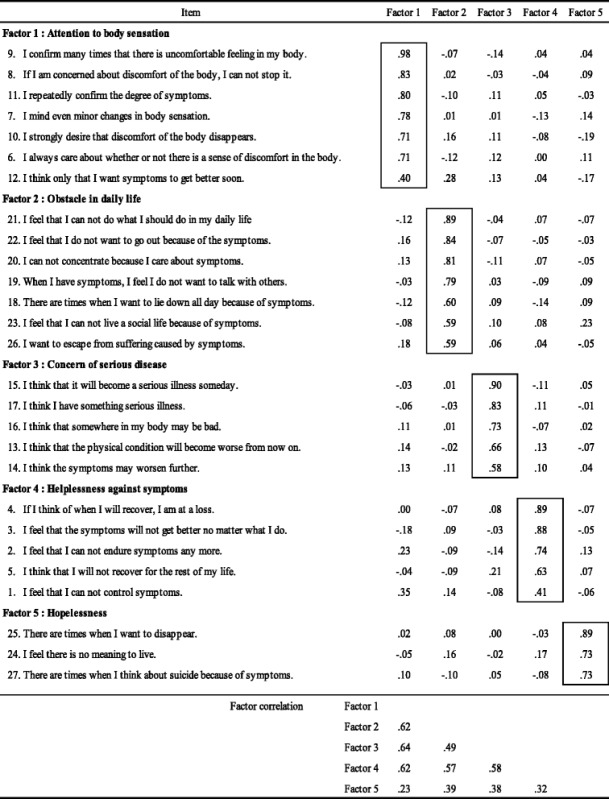
Results of factor analysis on somatosensory catastrophizing scale

### Second part of the study

**Fig. 2:**
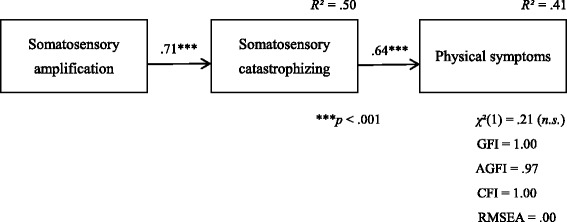
Results of covariance structure analysis of somatosensory amplification, somatosensory catastrophizing, and physical symptoms in the patient group

Firstly, the independent t-test between the patient group and the control group showed that the patient group scored significantly higher than the control group for all the variables (scores except SSAS: *p* < .001, SSAS score: *p* < .05). Secondly, we calculated Pearson’s correlation coefficients between all the variables. There were significant correlations between all the variables in both groups. To examine the effect of somatosensory amplification and somatosensory catastrophizing on physical symptoms, we conducted multiple regression analysis using the forced-on method. We used the SSCS and SSAS scores as independent variables and the MSC score as the dependent variable. For both groups, the SSCS score had a significant positive effect on the MSC score (patient group: *β* = .70, *p* < .01, healthy group: *β* = .27, *p* < .05), but the SSAS did not. We also conducted covariance structure analysis on both groups. We assumed multiple models of the influence of somatosensory amplification, somatosensory catastrophizing, and physical symptoms and adopted a model in which somatosensory amplification leads to somatosensory catastrophizing, from which physical symptoms arise. To evaluate the model, we used *χ* ^*2*^ (1) = .21 (*p* > .005, hereinafter called “ns”), GFI = 1.00, AGFI = .97, CFI = 1.00, RMSEA = .00 in the patient group, and *χ* ^*2*^ (1) = 3.22 (ns), GFI = .98, AGFI = .90, CFI = .97, RMSEA = .09 in the control group. The final model of the patient group is shown in Fig. 2.

We investigated the differences between the models of both groups by comparing a model with no equality constraints (Model 1) to a model that imposes equality constraints on all path coefficients (Model 2). We conducted simultaneous multi-population analysis and found that the Akaike information criterion (AIC) and Browne - Cudeck criterion (BCC) values were lower in Model 1. We calculated AIC = 23.42, BCC = 25.35 in Model 1 and AIC = 25.75, BCC = 27.29 in Model 2. Hence, we adopted Model 1. We investigated the differences between the path coefficients of the two groups by performing significant difference tests between the parameters. As ns = −1.40, there were no significant differences in the path coefficients from somatosensory amplification to somatosensory catastrophizing. There was a significant difference between the path coefficient from somatosensory catastrophizing to physical symptoms, as ns = −2.15 and *p* < .05.

## Discussion

In the first part of this study, we developed the SSCS, which was based on previous study [4]. We then assessed its reliability and validity. We focused on the effects of somatosensory amplification and somatosensory catastrophizing on physical symptoms. The resulting SSCS consists of 27 items relating to five areas. Our statistical analysis confirmed the reliability and validity of the SSCS. In previous study [4], somatosensory catastrophizing has been assumed to be an important factor influencing physical symptoms. However, as no standardized measurement scale was available, this assumption has not been tested empirically. In future studies, researchers will be able to use the SSCS to empirically study somatosensory catastrophizing.

In the second part of this study we investigated the relationship between somatosensory amplification, somatosensory catastrophizing and physical symptoms by analyzing and comparing the results from a patient group and a control group. The patient group exhibited significantly more somatosensory amplification and somatosensory catastrophizing than the control group. The model “somatosensory amplifcation”, via “somatosensory catastrophizing”, to “physical symptoms” was adopted for both groups. We believe this model to be valid as somatosensory amplification is a semi-inherent perceptual property. This model suggests that symptoms are exacerbated by catastrophic thinking and by a focus on the amplified body sensation.

Our model leads us to propose that intervention methods to alleviate physical symptoms should focus on eliminating catastrophic thought. For example, it is possible to prevent exacerbation of physical symptoms by giving psychoeducational therapy to a person suffering from high somatosensory amplification. It is important not to catastrophize when experiencing unusual bodily sensations. It may be possible to alleviate physical symptoms by applying techniques that aim to prevent catastrophic thoughts about bodily sensations. Such techniques include cognitive restructuring and intervention strategies such as mindfulness and attention training, which aim to prevent excessive attention being directed to bodily sensations.

We investigated whether our model confirms any differences between the patient group and the control group. The influence of catastrophic thought on physical symptoms was stronger in the patient group than in the control group. In particular, the participants in the patient group were found to experience physical symptoms as a result of catastrophic thought more readily than those in the control group. It is possible that the patient group was hypersensitive to thought.

In future studies, we intend to first investigate which background characteristics contribute to somatosensory catastrophizing. Catastrophic thought is considered to be a type of automatic thought based on a core belief that exists in the background, and it is thought that as a background for catastrophic thought to body sensation, the presence of belief about health such as "I'm not confident in my health, but should be healthy" or "It is easy to lose physical condition, but I should be in good condition as possible" is assumed. For this reason, we need to give further consideration to the relationship between beliefs concerning health and catastrophic thought.

Secondly, we must consider the physiological mediators in the process by which psychological phenomena such as catastrophic thought lead to the exacerbation of physical symptoms. Representative factors that connect psychology to physical symptoms include the nervous, endocrine, and immune systems. In addition to these systems, the fascia system and intestinal environment should be investigated. These are susceptible to psychological conditions and are thought to play a significant role in the psychological impact of physical symptoms. For example, it may be informative to investigate myofascial pain syndrome (MPS), which is a pain disorder that predominantly causes muscle pain [15]. In addition to pain, autonomic symptoms such as abnormal sweating and vasoconstriction; symptoms related to immune function such as skin infections; various symptoms such as equilibrium disorder and dizziness; coordination disorders, etc.; and musculoskeletal disorders such as headache, temporomandibular disorder, carpal tunnel syndrome, etc. have been raised as possible contributing factors [16]. MPS is among the disorders that are currently considered to be unexplained [17].

In contrast, fascia is rarely assumed to be a source of symptoms, and doctors have not received education on this subject. As a result, numerous unnecessary medications and surgeries have been given to patients suffering from fascia [17]. There have been reports relating MPS to psychological symptoms. For example, emotions such as anxiety and tension have been reported to have a negative effect on muscle tone and to reduce local blood flow due to hyperplasia of sympathetic ganglia. This causes symptoms to worsen [17]. Additionally, those diagnosed with MPS tend to score highly for neurosis on the Cornell medical index (CMI) and the Minnesota multiphasic personality inventory (MMPI) [18]. Hence, MPS is considered to have a high degree of affinity with physical symptoms.

Numerous studies have shown that there is a relationship between the intestinal environment and the brain. Hence, disturbing the balance of the intestinal bacterial flora leads not only to symptoms related to the digestive system but also affects the entire body, including the central nervous system. A person’s intestinal environment is also affected by their psychological state. Negative emotions and stress disturb the balance of the intestinal bacterial flora and the intestinal permeability increases (the barrier function decreases). As a result, intestinal bacteria and their components are thought to migrate into the blood, causing symptoms such as inflammatory reactions.

**Fig. 3:**
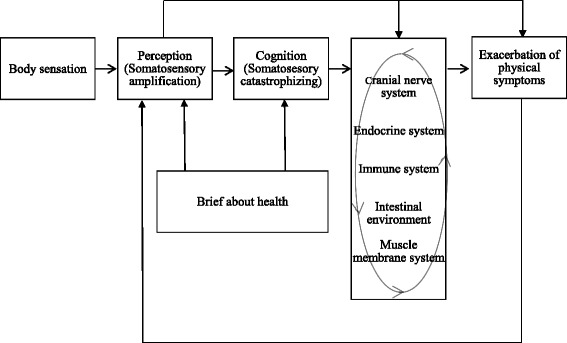
A theory of mediation factors and the processes of physical symptom exacerbation

The mediating factors and process by which physical symptoms are exacerbated are shown in Fig. 3. We hope that the physical and physiological factors that mediate between psychological states and physical symptoms will be investigated in more detail and that somatosensory amplification and somatosensory catastrophizing will be considered in these studies.

## Conclusions

In conclusion, we have developed the SSCS, which gives us a means of empirically measuring somatosensory catastrophizing. We also clarified the relationship between somatosensory amplification, somatosensory catastrophizing, and physical symptoms. In the future, we hope to research intervention techniques to mitigate the impact that somatosensory catastrophizing has on physical symptoms. Further research is required to determine the physical and physiological factors that mediate between psychological states and physical symptoms.

## Additional file

Additional file 1: Appendix. Somatosensory catastrophizing scale. (DOCX 26 kb)

## Abbreviations

AIC: Akaike information criterion
BCC: Browne - Cudeck criterion
MPS: Myofascial pain syndrome
MSC: Medical symptom checklist
PCS: Pain catastrophizing scale
SHAI: Short health anxiety inventory
SSAS: Somatosensory amplification scale
SSCS: Somatosensory catastrophizing scale
