# Supplementary material for: Relationships between catastrophic thought, bodily sensations and physical symptoms
Source: Biopsychosoc Med. 2017 Nov 8;11:28. doi: 10.1186/s13030-017-0110-z (PMC5678578; doi:10.1186/s13030-017-0110-z)
Supplement: Additional file 1: — Appendix. Somatosensory catastrophizing scale. (DOCX 26 kb) [file 13030_2017_110_MOESM1_ESM.docx]

**Appendix : Somatosensory catastrophizing scale**
